# Fern Spore Longevity in Saline Water: Can Sea Bottom Sediments Maintain a Viable Spore Bank?

**DOI:** 10.1371/journal.pone.0079470

**Published:** 2013-11-04

**Authors:** G. Arjen de Groot, Heinjo During

**Affiliations:** 1 Animal Ecology, Alterra – Wageningen UR, Wageningen, The Netherlands; 2 Ecology and Biodiversity, Institute of Environmental Biology, Utrecht University, Utrecht, The Netherlands; Roehampton university, United Kingdom

## Abstract

Freshwater and marine sediments often harbor reservoirs of plant diaspores, from which germination and establishment may occur whenever the sediment falls dry. Therewith, they form valuable records of historical inter- and intraspecific diversity, and are increasingly exploited to facilitate diversity establishment in new or restored nature areas. Yet, while ferns may constitute a considerable part of a vegetation’s diversity and sediments are known to contain fern spores, little is known about their longevity, which may suffer from inundation and - in sea bottoms - salt stress. We tested the potential of ferns to establish from a sea or lake bottom, using experimental studies on spore survival and gametophyte formation, as well as a spore bank analysis on sediments from a former Dutch inland sea. Our experimental results revealed clear differences among species. For *Asplenium scolopendrium* and *Gymnocarpium dryopteris*, spore germination was not affected by inundated storage alone, but decreased with rising salt concentrations. In contrast, for *Asplenium trichomanes* subsp. *quadrivalens* germination decreased following inundation, but not in response to salt. Germination rates decreased with time of storage in saline water. Smaller and less viable gametophytes were produced when saline storage lasted for a year. Effects on germination and gametophyte development clearly differed among genotypes of *A. scolopendrium*. Spore bank analyses detected no viable spores in marine sediment layers. Only two very small gametophytes (identified as *Thelypteris palustris* via DNA barcoding) emerged from freshwater sediments. Both died before maturation. We conclude that marine, and likely even freshwater sediments, will generally be of little value for long-term storage of fern diversity. The development of any fern vegetation on a former sea floor will depend heavily on the deposition of spores onto the drained land by natural or artificial means of dispersal.

## Introduction

As large parts of the earth surface consists of freshwater or sea water, plant diaspores regularly end up in the water. Although diaspores of species suited for hydrochory may be transported over the water for a long period of time (e.g. [1]), eventually most will sink to the bottom, where they will end up in the river, lake or sea bottom sediment. These sediments may therefore contain a reservoir of viable diaspores, from which species may germinate and establish whenever the sediment falls dry. In this way, sediments can play an important role in the local maintenance of biodiversity. Long-term survival of unfavorable circumstances via a robust life stage in the sediment has been shown both for animals (e.g. copepod egg banks [2]) and plants (e.g. episodic bryophytes [3]), and represents a strategy to deal with climatic fluctuations or extreme climatic events [4]. Diaspore banks also have the potential to harbor an important historical record of the local species and genetic diversity, which can be exploited to recover lost or degraded vegetations [5,6]. Aquatic sediments are increasingly applied for the restoration of riparian and shoreline vegetations (e.g. [7]). At the same time, the diaspore bank may accumulate diaspores of new or locally rare species and allow them to establish when a dry lake or river bed is (re)vegetated (e.g. [8]). 

Whether, and which, species will eventually be able to colonize depends not only on abilities for dispersal, germination and establishment, but also on the ability of their diaspores to survive sufficiently long to form a persistent diaspore bank in the sediment [9]. For angiosperms, seed longevities are known to vary strongly between species and environments [10]. Much less is known for spore plants, however. The presence of fern spores in freshwater and marine sediments has been reported in various paleolimnological studies (e.g. [11,12]), but viability of these spores remained untested. Recently, the potential of ferns and bryophytes to produce a persistent soil spore bank has gained more attention [3,6,13-16], but such studies were limited to terrestrial environments. We do not know of any study that attempted to test whether fern spores survive in waterlogged sediments. Experimental studies showed wet storage to be more effective for spore conservation than dry storage [17,18], but this concerned storage on a humid agar medium rather than spores being fully inundated. 

Long-term survival of diaspores in sea bottoms has generally been considered negligible for physiological reasons, as longevity is likely impacted by saline conditions at least for non-halophytic species. Several investigators showed that seeds of glycophytic plants survive for only a short period of time at seawater salinity levels (e.g. [19]). Apart from cell burst due to osmotic stress, enduring exposure to high salt concentrations often also results in cell damage due to oxidative stress [20]. Even when seeds remain viable until the soil falls dry, the salinity of the substrate may limit germination and reduce seedling growth (e.g. [21,22]). As salt tolerance depends on specific physiological responses such as ion exchange capacities, tolerance levels are at least partly genetically based and thus may vary between species and genotypes (e.g. [9]). While the salt tolerance of seeds and seedlings has been described for a variety of species, it has rarely been tested for spores and gametophytes of cryptogams. Warne et al. [23] tested the effects of salinity on mutants of *Ceratopteris richardii* and then studied the physiology of a salt-tolerant mutant. Bogdanović et al. [24] showed reduced chlorophyll content in gametophytes of various ferns and mosses when grown on very saline media (>100 mM NaCl). Reduced germination, gametophytic growth and gametangial development on saline substrates have been found even for the halophytic species *Asplenium marinum* [25], *Acrostichum aureum* and *Acrostichum danaeifolium* [26,27]. We did not find any study that investigated the effect of saline incubation on spore viability. 

The survival and germination of spores under saline conditions is of particular importance for nature development in the Netherlands, where large areas of sea bottom have been drained to create new polder lands. Various wetland and forest nature areas have been developed in these polders, which represent unique opportunities to study the sources of species and genetic diversity at large scales and with sufficient replication [28]. Some planted forests in the IJsselmeer polders, areas of artificial land created in the twentieth century in a former inland sea (Zuiderzee) in the centre of the Netherlands, already harbor a surprising diversity of fern species, including a number of rare calcicoles of which the nearest populations were located up to 250 km away at the time of first colonization [29]. Previous authors studying the colonization of the Dutch polders by these rare ferns [29,30] assumed that all viable spores must have arrived by wind after the sea bottom was drained, largely neglecting the potential presence of viable spores in the sea bottom sediment. Yet, numerous spores will have reached the inland sea either by wind dispersal or by transportation along the rivers that flow into it (e.g. [1]), and many of them will have ended up in the local sediments. 

In this study we attempted to test the potential of fern spores to survive in a sea bottom and to yield viable gametophytes in occasions when the sediment falls dry. We tested this for three different species and genotypes that have been able to colonize the Dutch polder lands. Aset of experiments was performed to test for spore germination and gametophyte fitness after inundated storage and storage in salt water at varying concentrations and varying periods of storage. We also tested the effect of substrate salinity on germination and gametophytic growth. Furthermore, to directly confirm the presence of viable spores in sea and lake bottom sediments, we conducted a spore bank analysis on sediment cores from the bottom of the former inland sea.

## Materials and Methods

### Study area and study species

In 1932, the inland sea in the centre of the Netherlands, the Zuiderzee, was dammed from the North Sea and became a freshwater lake. From 1942 onwards, polders were created in the eastern part of the lake. The rest of the lake still exists and is referred to as the IJsselmeer. The polder lands are referred to as the IJsselmeer polders. Here, we use the IJsselmeer area as a case study to test the potential of fern spores to survive under long-term saline inundated conditions. Apart from a spore bank experiment based on sediment cores from the bottom of the IJsselmeer, we collected spores from three species occurring in the Dutch IJsselmeer polders, to be used in two different storage experiments: *Asplenium trichomanes* ssp. *quadrivalens, Asplenium scolopendrium* and *Gymnocarpium dryopteris*. All three species are rare in the Netherlands, but were able to establish multiple populations in the IJsselmeer polders since the 1970’s. Previous studies have indicated that the spore rain reaching the IJsselmeer area is sufficiently dense to result in spore reservoirs of considerable size [29-31].

We collected fertile fronds in October 2006 (experiment 1) and 2007 (experiment 2) in the Kuinderbos, the most species-rich forests in the IJsselmeer polders [29]. *Asplenium trichomanes* and *A. scolopendrium* are legally protected species in The Netherlands. Permission for collection and possession of this material was granted by the responsible ministry (Ministry of Economic Affairs; license number FF/75A/2009/066a). No permission was required for collection of *G. dryopteris*, which is no protected species. The forest’s owner, the State Forestry Service, granted permission to work on its property.

### Experiment *1*: effects of wet storage and salinity

Collected fronds were air-dried and spores were extracted by tapping on the dry fronds above a white paper sheet. Spores of each of the three species were then stored at six different conditions: dry, inundated in normal tap water and inundated in normal tap water to which four different levels of NaCl were added: 1.0, 2.5, 5.0 and 10.0 g L^-1^ (^≈^ 17.0, 42.8, 85.6 and 171.2 mM). Freshwater contains <0.5 g L^-1^ [32]. As our study was originally intended to test the potential for spore survival in the sediments of the former Zuiderzee inland sea, we took the historical concentration in these sediments as a maximum. True marine sediments contain higher salt concentrations (up to 37 g L^-1^ [32]). Storage treatments were created by putting 0.01 grams of spores in each of six separate vials, leaving one vial dry and adding 0.5 ml water of different salt concentrations to the other five vials using a sterile pipette. During storage, all vials were placed in the dark at 4 °C [33]. Air humidity in the dry vial was below 25% at ambient room temperature, but may in fact have become considerably higher when vials were transfered to 4 °C. Thus “dry storage” in our experiment refers not to a total lack of water, but to air-dry conditions. No condense water was observed in the dry vials. After one month of storage, the spores were sown onto petri dishes containing a medium consisting of Parker’s macronutrients and Thompson’s micronutrients [34] solidified with Gelrite® (5.0 g L^-1^). Due to practical constraints, we were unable to store the spores for more extended periods. Spores were sown by first resuspending the spores in the vial by vortexing and then spreading a fixed amount of fluid (100 μL) on the surface of the culture medium, under sterile conditions in a flow chamber. In case spores were stored in saline water this procedure added a small amount of salt to the culture medium. Yet, the resulting rise of salinity was assumed to be negligible. To determine the effect of a saline substrate on germination, spores from each storage treatment were subjected to two different culture treatments by adding different levels of NaCl to the liquid medium prior to sterilization: 0.0 g L^-1^ (control) and 2.5 g L^-1^ (simulating brackish soil). Each combination of storage and culture treatments was replicated three times per species. Spore sowing densities (number of spores per cm^2^) were measured by counting the number of spores in 25 randomly positioned squares of 2x2 mm. Petri dishes were sealed with Parafilm® and placed in a growth cabinet at 20 °C and a photoperiod of 12h dark and 12h light, with a light intensity of ±50 μmol photons of PAR m^-2^ s^-1^. After 3 weeks, the germination percentage in each petri dish was determined by randomly checking 100 spores on the dish and counting how many had germinated [18]. A spore was considered germinated if the spore wall had ruptured and a first cell had started to emerge [18,35]. After nine weeks, gametophyte size was determined for 10 individuals per dish by making a photograph through a stereomicroscope and transferring pixel amounts into a metric area measure (mm^2^) using image analysis software (*ImageJ* [36]). ANCOVA tests in SPSS 19.0 (IBM Corp., New York), using spore sowing density as a covariate, were used to test for significant differences between treatments in germination percentage and gametophyte size. Subsequently, Tukey post-hoc analyses were performed to analyze pairwise differences between treatments with different salt concentrations. All germination percentages were arc-sine transformed prior to analysis [18].

### Experiment *2*: long-term storage in saline water

Spores were collected from fertile fronds of three different sporophytes of *A. scolopendrium*. Each sporophyte originated from a different population in the forest and constituted a different genotype [31]. Spores from each genotype were poured into 24 vials (Eppendorf®, 1.5 ml), so that each vial contained 0.01 grams of spores. Spores in the vials were subjected to one out of two different storage treatments: storage in saline water (i.e. 0.5 ml of a 10.0 g L^-1^ NaCl solution was added to the vial and the vial was vortexed to suspend the spores in the fluid; N=12) and dry storage (i.e. air-dry storage, vials were closed without addition of saline water; N=12). 

Vials were stored in the dark at 4 °C. At four moments in time (after 3, 12 and 24 months of storage) three vials with wet stored spores and three control vials were randomly selected and spores were sown onto separate petri dishes containing the same medium as described above (but without addition of NaCl). Initial germination rates before storage were tested by sowing similar amounts of spores onto three petri dishes at the start of the experiment. Procedures for sowing and culturing, as well as estimations of sowing densities, germination rates and gametophyte sizes resembled those in experiment 1. A repeated measures analysis in SPSS 19.0, using time as within-subjects factor, was used to test for significant differences between treatments in germination percentage (arc-sine transformed values). For the 12 months storage treatment, we also assessed gametophyte survival per dish after nine weeks of cultivation and measured the size of ten randomly selected surviving gametophytes. Survival was measured as the percentage of 100 randomly selected gametophytes that was still alive; a gametophyte was defined as alive in case one or more green cells were visible. The size of the surviving gametophytes was measured as in experiment 1. Significance of the treatment effects on survival (arc-sine transformed values) and size were assessed using GLM models in SPSS 19.0.

### Spore bank analysis

**Figure 1:**
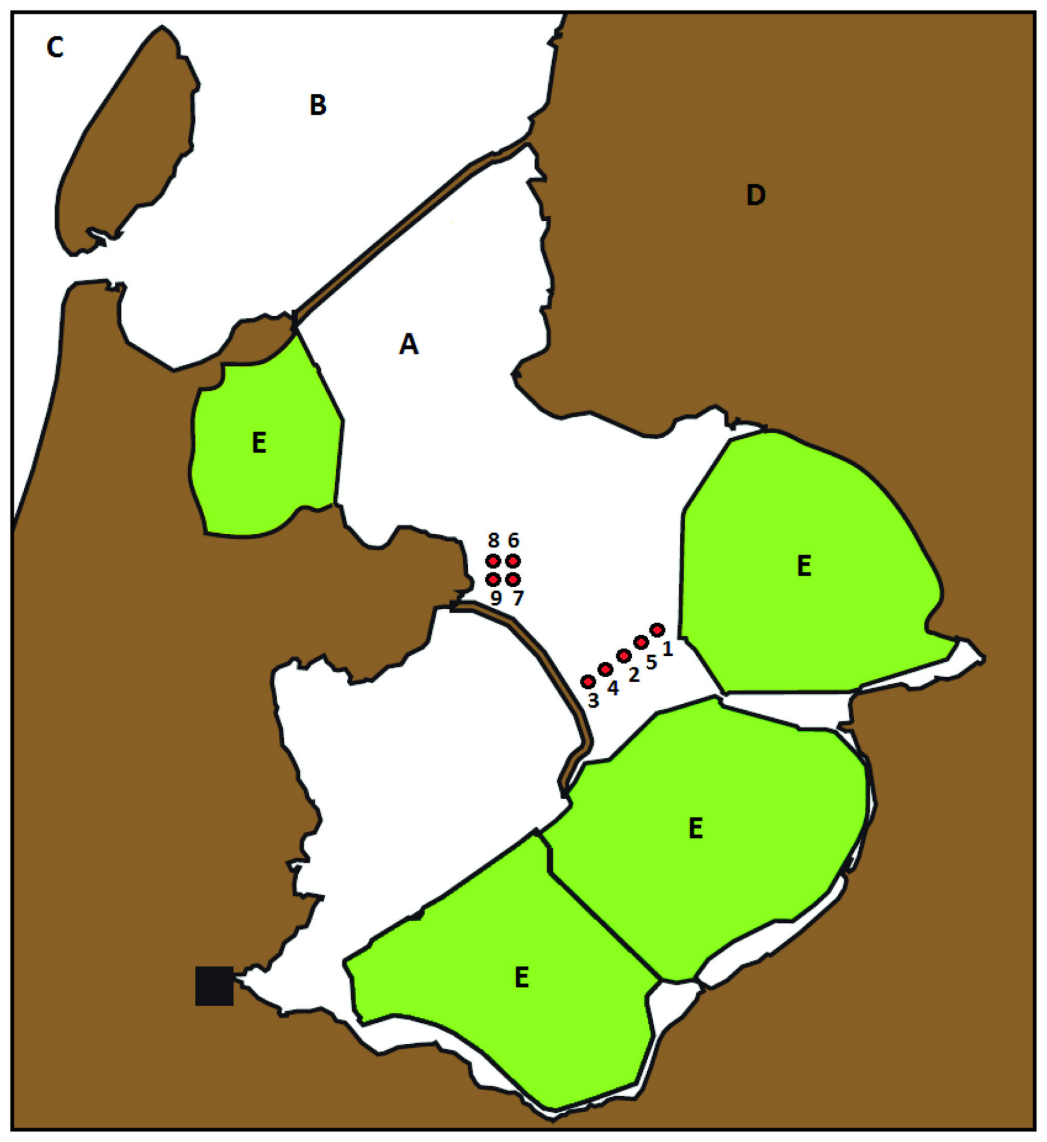
Drilling locations for spore bank analysis of nine lake sediment cores. A = lake IJsselmeer, B = Wadden Sea, C = North Sea, D = Dutch mainland, E = IJsselmeer polders. Black square = Amsterdam.

In February 2008, nine sediment cores were drilled in two regions of the IJsselmeer (Figure 1), to check for the presence of viable spores. The IJsselmeer area is state property, and no legal permission was required for the collection of sediment samples from these waters. The obtained cores measured up to 25cm in length, and consisted of a top layer of black gyttja, representing the lake sediment, on top of coarse-grained yellow sand with mollusk shells, originating from the time the area was still a saline inland sea. The two layers are separated by a thin layer of fine grey sand, that was deposited during the brackish transition period (1932-1938 [37]). Based on palynological analysis of a sediment core from a nearby part of the lake, Cremer et al. [37] estimated the local mean sedimentation speed to be ±0.5 cm year^-1^. As depth of the freshwater sediment layer was much less than the 35cm expected to establish since 1938, either the sedimentation speed must have been lower, or part of the sediment must have flushed away. Yet, we used this rate of 0.5 cm year^-1^ to calculate a rough estimate of the minimal age of the sand at a certain vertical distance from the top of the sediment core. The marine sediment below the transition layer is known to date from before 1932, and any spores in this layer must have experienced saline conditions. Each core was subdivided in slices of 1 cm thick, which were then subjected to a spore bank analysis. Each slice was homogenized and spread out on top of a 2 cm layer of moist, sterilized sand in a 8x8cm plastic box with a closed, transparent lid. All boxes were placed in a randomized order in the greenhouse of the Botanical Gardens of Utrecht University to allow germination of any viable diaspores. For exact culturing conditions see 38. For three months, the boxes were checked once every two weeks for fern gametophytes or bryophytes. Fern gametophytes were harvested to be identified via DNA barcoding (see 39 for the DNA barcoding methodology). Mosses were cultured in the same boxes until they could be identified morphologically. Herbarium vouchers of the mosses were deposited in the Dutch National Herbarium (L). Because of their small size, DNA barcoding of the fern gametophytes involved destructive extraction, yet DNA extracts are available from the molecular laboratory of the Ecology & Biodiversity Group, Utrecht University.

## Results

### Experiment *1*: effects of wet storage and salinity

No dark germination was observed during spore storage. Germination rates were high in all treatment and species combinations, ranging from 83-100%. The effect of inundated storage differed between the three study species. When dry-stored spores were compared to spores stored in tap water without additional NaCl (Table 1), no effect on germination or gametophyte growth was observed for *A. scolopendrium*. Spores of *G. dryopteris* germinated almost equally well in both treatments (even though the 2% difference was statistically significant), but wet-stored spores resulted in much larger gametophytes (Table 1). A similar effect on gametophyte size was observed in *A. trichomanes* subsp. *quadrivalens*, but germination percentage decreased after wet-storage. 

**Table 1:** Difference in germination percentage and gametophyte size following either dry or (non-saline) inundated spore storage.

	**Germination percentage**				**Gametophyte size**			
Species^1^	Dry	Inundated	F	P	Dry	Inundated	F	P
*ASPS*	94 ± 1.7	94 ± 1.6	0.245	0.632	1.68 ± 0.2	1.68 ± 0.8	0.01	0.947
*ASPT*	93 ± 1.7	82 ± 3.8	14.87	0.004	2.62 ± 0.4	3.93 ± 0.8	5.15	0.058
*GYMD*	97 ± 0.7	99 ± 0.8	12.12	0.007	1.07 ± 0.2	1.81 ± 0.2	10.82	0.013

^1^ *ASPS* = *Asplenium scolopendrium*, *ASPT* = *Asplenium trichomanes* subsp. *quadrivalens*, *GYMD* = *Gymnocarpium dryopteris*.

Means and standard errors are given for both spore germination percentage and gametophyte size (mm^2^) after spores had been subjected to the storage treatments for one month, along with the F-statistic and tested significance (P) of ANCOVA tests on the difference between treatments .Sowing density during germination was treated as a covariate.

**Figure 2:**
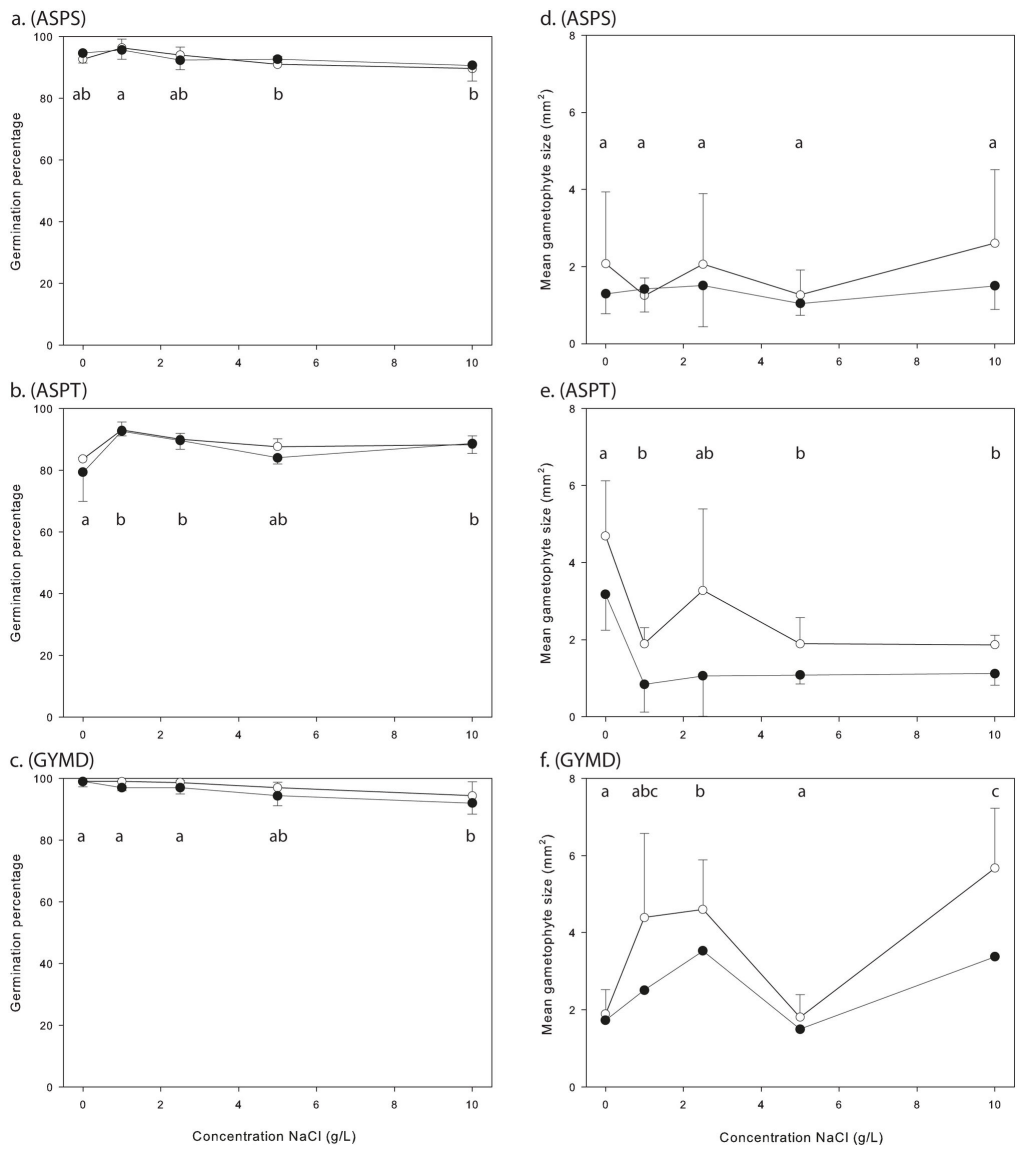
Effect of salt concentration during wet spore storage and during cultivation. Figure *a*, *b* and *c* show effects on spore germination; Figure *d*, *e* and *f* show effects on gametophyte size. Values on the X-axis represent salt concentrations in the storage medium. Results of cultivation on control and saline culture media are presented as separate lines (empty circles = control medium; filled circles = saline medium). The letters a-c in each graph represent the results of a post-hoc analysis testing for differences among salt concentrations: a difference between two salt concentrations is only significant if they do not share the same letter. Figure *a* and *d* = *Asplenium scolopendrium* (*ASPS*), Figure *b* and *e* = *Asplenium trichomanes* subsp. *quadrivalens* (*ASPT*), Figure *c* and *f* = *Gymnocarpium dryopteris* (*GYMD*).

The effects of increasing salt concentrations on germination and gametophyte growth are shown in Figure 2. For *A.scolopendrium and G. dryopteris* germination percentages were highest at low salt concentrations and lowest at high salt concentrations, although not all pairwise comparisons between treatments were statistically significant (Figure 2). For *A. trichomanes* subsp. *quadrivalens* spore germination increased when salt was added, but did not significantly change at higher salt concentrations. The overall effect of salt concentration during storage was significant in all three species (Table 2). No clear trends in gametophyte size were observed with increasing salt concentrations during storage (Figure 2). For *A. trichomanes* subsp. *quadrivalens* the presence of low levels of NaCl during storage already resulted in significantly smaller gametophytes, but further increases in concentration did not affect gametophyte size.

**Table 2:** Results of ANCOVA tests on the effect of salinity during one month of wet storage, the effect of salt content in the culturing substrate, and their interaction.

			**Storage salt concentration**		**Salt culture medium**		Storage*medium		**Spore density (covariate)**	
	**Variable**	**Species**	F	P	F	P	F	P	F	P
	Germination percentage	*ASPS*	4.01	0.012	0.18	0.671	0.41	0.797	0.00	0.936
		*ASPT*	9.65	0.000	0.91	0.351	0.53	0.713	0.40	0.531
		*GYMD*	5.30	0.003	3.69	0.073	0.27	0.893	0.00	0.940
	Gametophyte size	*ASPS*	0.58	0.684	2.06	0.680	0.83	0.524	5.17	0.035
		*ASPT*	6.97	0.001	10.77	0.004	0.49	0.744	0.94	0.344
		*GYMD*	8.43	<0.001	7.84	0.011	1.55	0.227	1.31	0.266

Spore density during germination was treated as a covariate. F-statistic and tested significance (P) are given for each treatment. See Table 1 for species acronyms.

The presence of salt in the cultivation medium did not affect germination rates (almost overlapping lines in Figure 2a-c), but did have a clear effect on gametophyte size (Figure 2d-f). Gametophytes of *A. trichomanes* subsp. *quadrivalens* and *G. dryopteris* were significantly smaller on a saline substrate, irrespective of the preceding storage treatment (Figure 2, Table 2). The same effect seemed present for *A. scolopendrium*, but was not significant due to large variation among replicates. 

### Experiment *2*: long-term storage in saline water

**Figure 3:**
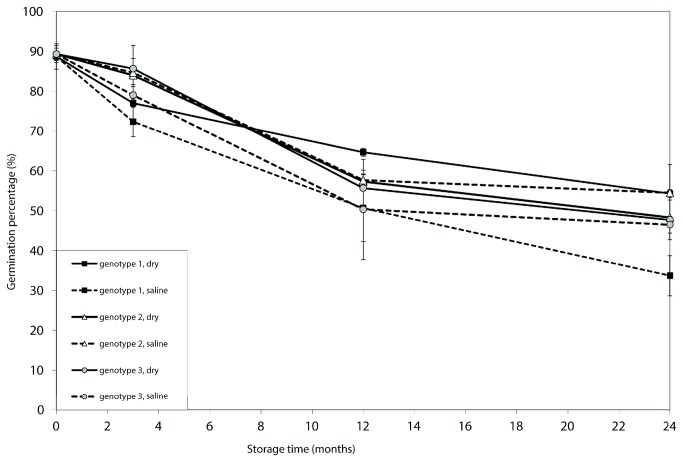
Germination success (percentage of germinated spores) after 0, 3, 12 and 24 months of storage. Different lines depict different combinations of parent genotypes of *A. scolopendrium* and storage treatments (see legend).

Germination percentages significantly dropped over time for all genotypes (Figure 3; Table 3). Yet, even after 24 months of saline storage, 34-55% of the saline stored spores still germinated. The effect of storage conditions clearly depended on the genotype of the parent plant (significant interaction in Table 3). Both genotype 1 and 3 showed consistently lower germination when spores were stored in saline water than when spores were stored dry, although this effect was no longer significant for genotype 3 when spores were stored for 24 months. Spores of genotype 2 germinated equally well at both conditions when stored up to 12 months, and after 24 months germination success was higher in the saline storage treatment. No dark germination was observed during storage.

**Table 3:** Results of a repeated measures analysis on the effect of storage time, storage conditions (dry or saline water) and parent genotype, on germination percentage.

	Germination percentage	
**Variable**	F	P
Storage time	901.50	<0.001
Storage conditions	10.43	0.007
Genotype	4.26	0.040
Time * Conditions	4.59	0.053
Time * Genotype	1.67	0.229
Conditions * Genotype	5.87	0.017
Time * Genotype * Conditions	5.01	0.026

**Figure 4:**
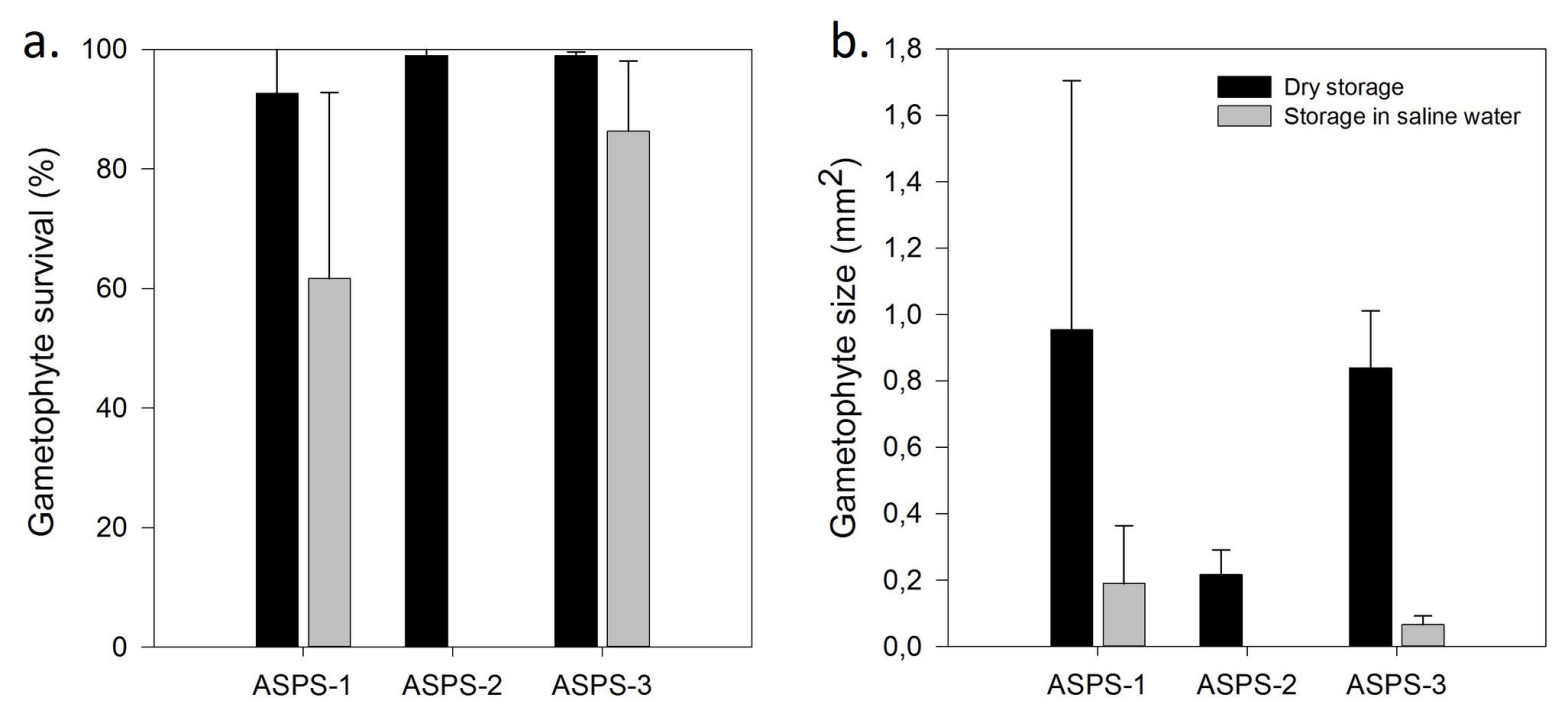
Survival and size of gametophytes grown from spores of three parental genotypes of *A. scolopendrium*. Survival rates are depicted in Figure 4a; gametophyte sizes are depicted in Figure 4b. Genotypes are coded ASPS-1 to ASPS-3, and were subjected to both dry storage or storage in saline water.

Gametophytes originating from spores stored for 12 months in saline water showed significantly lower survival rates than those from spores stored under dry conditions (Figure 4a; F= 16.56 P=0.002). This effect was present in all genotypes, but differed between them (interaction: F=4.29; P=0.042). The most dramatic effect was visible in genotype 2, for which all gametophytes that originated from spores stored in saline water had died after nine weeks of cultivation, while all gametophytes from dry stored spores were still alive. In the other two genotypes, spores stored in saline water resulted in smaller gametophytes (Figure 4b), although this effect was only significant in genotype 3 (F=64.68, P=0.001). 

### Spore bank analysis

Two fern gametophytes, both identified as *Thelypteris palustris*, emerged from the sediment of two cores drilled from different parts of the lake (Table 4, Figure 1). One was obtained from the top centimeter of the sediment, the other came from 5-6cm depth, corresponding with a minimal age of 10 years. Both gametophytes remained of small size and pale color, and failed to produce gametangia. Bryophyte shoots of four different species were observed in a total of three samples (Table 4). Yet, in all cases the sample contained multiple shoots, that seemed to originate from independent protonema. Interestingly, the only sample from which more than one species emerged (*Marchantia polymorpha, Funaria hygrometrica* and *Leptobryum pyriforme*) came from a depth of 13-14 cm, corresponding to a minimal age of 26 years. All samples for which germination was recorded originated from the freshwater lake sediment, no germination was observed for samples of the marine sediment layer.

**Table 4:** Species found in sediment samples from the IJsselmeer lake bottom.

Core	Depth (cm)	Minimal age (years)	Species	Abundance
1	5-6	10	*Thelypteris palustris*	1
2	1-2	2	*Bryum* sp.	2
4	13-14	26	*Marchantia polymorpha*	2
			*Funaria hygrometrica*	3
			*Leptobryum pyriforme*	3
8	0-1	0	*Thelypteris palustris*	1
9	3-4	6	*Leptobryum pyriforme*	3

“Minimal age” refers to the estimated minimal age of the slice of sediment in which the species was detected (see methodology). The abundance of bryophyte shoots and fern gametophytes in the culture box is presented using the following categories: 1 = one shoot/gametophyte; 2 = two to ten shoots; 3 = >10 shoots.

## Discussion

In recent years, many attempts have been made to restore nature areas in order to conserve and promote levels of natural diversity. Yet, restoration measures regularly failed to yield the expected biodiversity, since many characteristic species did not return to the local vegetation (e.g. [40]). As dispersal abilities often seem to be a limiting factor [41], the presence of diaspores in local of nearby soils may be of considerable importance to the establishment of a diverse vegetation, both at the species and population genetic level. Although long-distance dispersal of spores may be sufficiently common to allow rapid establishment of both inter- and intraspecifically diverse fern vegetations [31], soil banks may also importantly contribute to the local diversity (e.g. [5,6]). In this study, we tested the potential of sea bottom sediments to aid in the establishment of a diverse vegetation after the water has been withdrawn. This potential has been confirmed for freshwater sediments [7], but has never been tested before for saline environments. 

For colonization from a former sea bottom to be successful, spores should be able to endure both inundated (anoxic) conditions and high NaCl-concentrations for a long period of time to allow germination when conditions change (1), as well as to produce a gametophyte capable of reproduction (2). Moreover, germination and gametophyte growth must be possible under saline conditions (3). We tested each of these aspects of colonization under experimental conditions, and then checked the overall potential for colonization from actual sediment samples via spore bank analysis. The results of both types of studies consistently suggested limited colonization potential, at least for the non-halophytic fern taxa occurring in the Netherlands. Below, we will use our results to discuss the three aspects of colonization mentioned above.

### Survival and germination of spores subjected to long-term (saline) inundation

From a total of nine soil cores, we retrieved only two viable fern spores, both of a species that commonly occurs in the wetlands surrounding the lake (*Thelypteris palustris*). Previous studies of spore banks in the nearby polders indicated that the spore rain reaching the IJsselmeer area is sufficiently dense to result in spore reservoirs of considerable size [30]. We know that this may also be true for the IJsselmeer lake bottom sediment, as a local paleobotanical study by [42] retrieved spores of at least six fern genera from a single sediment core (including rare taxa such as *Ophioglossum* and *Botrychium*). Although we did not check for non-viable spores, and water currents may result in very uneven spatial distributions, at least some of our cores likely contained much higher numbers of spores than those that germinated from our lake and marine sediments (two and zero spores respectively). Thus, fern spores seem not to survive in the marine sediment, and only to a limited extent in the lake sediment. The same is likely true for bryophytes, of which also only few shoots of a limited number of species emerged from our sediment cores. A few previous studies have investigated the survival of bryophyte propagules under inundated conditions [43], and showed that capacities differ among species. Some of them (e.g. *Physcomitrium sphaericum*) seemed to be adapted for long-term survival in sediments [44]. 

The results obtained from our lake sediment cores are in line with those of a diaspore bank analysis conducted around the same time for the soils underneath the new forests in the IJsselmeer polders [30,39]. While many species emerged from the top layers, the diversity and abundance of emerging ferns, bryophytes and phanerogams dropped rapidly towards the lower layers, which again represent the former lake and sea bottom. Although such skewed distributions with depth are commonly observed in diaspore banks (e.g. [13]), the former conditions in these soils may have contributed to the loss of diversity. 

The descriptive results outlined above are also in line with our experimental results for *A. scolopendrium*, which showed spores of at least one of the three genotypes (genotype 3) to germinate less following long-term inundation in water containing salt concentrations approaching those of sea water. In reality, germination might be affected even more, as true marine sediments have considerably higher salt concentrations. Yet, two remarks must be made. First, the difference between treatments was highly variable among genotypes, and saline storage even seemed to have a positive effect in genotype 2 after two years of storage. Secondly, in all genotypes the germination of dry-stored spores also dropped substantially over the two-year period, perhaps due to the fact that spores in our dry-storage treatment may in fact have experienced an air humidity that is suboptimal for spore survival (RH>50 [45]). Yet, our purpose was to compare conditions in inundated soils to conditions in temperate terestrial soils, which will likely never be completely dry. 

The presence of high salt concentrations in the water generally appeared to be more detrimental to spore survival than inundation per se. In both *A. scolopendrium* and *G. dryopteris*, germination was not reduced by inundation in salt-free water, but was reduced (to some extent) at enhanced NaCl concentrations. Yet, *A. trichomanes* subsp. *quadrivalens* showed the opposite result: the observed reduction in germination success when inundated in pure tap water disappeared when salt was added to the water. Whether this species-specific variation in salt-tolerance is consistent within and among genotypes, and whether it has any physiological basis, remains to be tested. Future tests should also include inundation for longer time periods (>1 month), to check whether at such time scales even inundation in salt-free water reduces spore viability. A previous study by Quintanilla et al. [18] showed, however, that wet storage might in some species indeed result in similar, or even enhanced spore conservation.

It must be noted that both in our experiments and in the field study, we used germination capacity as a proxy for spore survival. By doing so, we may have underestimated survival, as previous studies have shown that saline conditions can induce long-term seed dormancy in halophytes [46]. No information is available on salt-induced dormancy in ferns, but the existence of dormancy in fern spore banks has been proposed in the past [16]. Future experiments should clarify this issue.

### Gametophyte development from spores subjected to saline inundation

Although no clear effect of storage conditions on gametophyte performance was observed after one month of spore storage, both the size and survival of gametophyte were clearly reduced when spores were subjected to long-term storage in salt water. This is consistent with our observations for the two gametophytes that emerged from the sediment cores, which were pale (low chlorophyll content), small and short-lived. Apparently, even in case germination is still possible, some salt-induced damage may occur at the long term and may put constraints on the production of normal gametophytes. Reduced gametophyte longevity may strongly limit colonization potential by reducing the period during which successful fertilization may occur. This is of special relevance in case spore densities are low and for species with limited selfing capacities [47]. Our results for *A. scolopendrium*, showing clear differences in the effect of spore storage conditions among genotypes, suggest the possibility of a genetic basis for tolerance to saline inundated storage. The death of all gametophytes that originated from spores of genotype 2 stored in saline water is unlikely caused by interfering environmental conditions during storage or cultivation, as it occurred across replicates. Yet, we used spores from only one plant per genotype, which means that an unknown factor affecting individual plants may have interfered (fronds of our ‘genotype 2’-plant might for instance have been infected with a pathogen, that survived on the spore walls and infested the gametophytes produced). It is interesting to see, however, that differences in effect size among genotypes were not the same with respect to gametophytic growth (Figure 4) and germination (Figure 3). This reinforces the idea that genotypic differences may play a role. 

### Germination and gametophyte development on a saline substrate

The few past studies that did investigate salt-tolerance of ferns all focused on the effect of direct exposure to salt stress during germination and gametophyte cultivation [23-27]. Although we focused on the effect of salt-stress endured by spores on future germination and gametophytic growth, direct effects are also relevant to investigations of colonization potential from sea bottom soils, as the absence of the sea water will not instantly remove all salt from the soil. In fact the soil water salinity is still brackish on many locations in the Ijsselmeer polder lands (0.5-1 g/L Cl^-^) and very high compared to the Dutch mainland [48].

While direct inhibition of germination on saline substrates is common among angiosperms [9], our results do not give any indication for such an effect in ferns. Yet, gametophytes were clearly affected, as they grew less fast when grown on saline media. Stronger effects in the gametophytic stage are not surprising, given their delicate structure (a single layer of cells, with no or only a thin cuticle [24]). Bogdanović et al. [24] also reported a lower chlorophyll content under saline conditions for *A. scolopendrium* and two other fern species. This may also have been the case in our experiment as some paleness was occasionally observed, but we did not consistently quantify chlorophyll contents.

### Vegetation development on former sea floors

Occasionally small parts of salinized sediment get exposed to the air and may form a substrate for new plant communities to develop. Around the world, mankind reclaims land from the sea. Large parts of the Dutch polder lands for example are in fact former sea bottoms, which now harbor valuable new nature areas [29]. Results from our spore bank experiment suggest that occasionally spores of some species may survive in the sediment and may aid to the establishment of a fern vegetation on such new lands. This will likely concern species that naturally occur in riparian (e.g. *Thelypteris palustris*; this study) or marine coastal conditions (e.g. *Asplenium marinum* and *Acrostichum* sp. [25-27]). Long-term storage experiments should be performed also for these species to test whether they are indeed better capable of surviving saline inundated conditions. We do not know of any studies on the spore longevity of halophytic or riparian pteridophytes.

 Still, our results suggest that at best only a small part of the diversity will be conserved in the sediment. In general, the establishment of a fern vegetation on former sea bottoms will almost entirely be driven by new (wind) dispersal, as was assumed by previous authors [29,30]. 

Ongoing changes in climate and land use may also result in local soil salinization, by shifting water levels or increasing evaporation. We conclude that such changed soil conditions may strongly affect the presence of local spore banks, which may affect the fern diversity in the aboveground vegetation at the long term [6].
